# The Influence of Elementary School Personnel’s Achievement Goal Orientation on CPR Education Immersion and Self-Management

**DOI:** 10.3390/ijerph23020260

**Published:** 2026-02-19

**Authors:** Tae-Young Moon, Hyeon-Ji Lee, Mi-Young Choi

**Affiliations:** Department of Paramedicine, Kangwon National University, 346 Hwangjo-Gil, Samcheok-City 25949, Gangwon-do, Republic of Korea

**Keywords:** achievement goal orientation, CPR education immersion, self-management, elementary school personnel, motivation

## Abstract

**Highlights:**

**Public health relevance—How does this work relate to a public health issue?**

- Bystander cardiopulmonary resuscitation (CPR) performed by school personnel is a critical determinant of survival in pediatric out-of-hospital cardiac arrest.
- Understanding motivational and psychological factors that influence engagement in CPR training is essential for strengthening school-based emergency preparedness.

**Public health significance—Why is this work of significance to public health?**

- This study demonstrates that achievement goal orientation is significantly associated with immersion in CPR education and self-management among elementary school personnel.
- Mastery-oriented motivation was identified as a key factor promoting sustained self-regulatory behaviors relevant to emergency response readiness.

**Public health implications—What are the key implications or messages for practitioners, policy makers and/or researchers in public health?**

- CPR training programs for school personnel should adopt mastery-oriented, learner-centered strategies to enhance engagement and long-term skill maintenance.
- Educational policies should incorporate motivational and self-regulation components, in addition to procedural instruction, to improve the effectiveness of school-based CPR training.

**Abstract:**

Despite mandatory cardiopulmonary resuscitation (CPR) training for elementary school personnel in South Korea, current programs are often delivered as uniform, compliance-oriented sessions that prioritize procedural completion over sustained engagement and self-regulated practice. However, little empirical evidence explains why some participants remain actively engaged and capable of self-managing CPR skills after mandatory training, while others do not. In this study, immersion in CPR education refers to learners’ cognitive and behavioral engagement during training, reflecting their concentration and active participation in learning activities. Accordingly, this study aimed to examine how achievement goal orientation influences CPR education immersion and self-management among elementary school educational officials. A survey was conducted from March to June 2024 with 150 teachers and administrative staff in Gangwon Province, South Korea. Data were analyzed using correlation and multiple regression analyses. The results showed significant positive correlations among achievement goal orientation, CPR education immersion, and self-management. Both self-goal and task goal orientations significantly increased CPR education immersion, whereas only task goal orientation positively influenced self-management. In addition, both cognitive and behavioral immersion significantly predicted self-management These findings suggest that mastery-oriented motivation is associated with deeper engagement during CPR training and stronger self-management, supporting motivationally informed instructional design rather than compliance-focused delivery.

## 1. Introduction

Out-of-hospital cardiac arrest (OHCA) remains a critical public health issue, and timely bystander cardiopulmonary resuscitation (CPR) is a key determinant of survival. Immediate CPR performed by laypersons has been reported to increase survival rates by two- to three-fold in OHCA situations. In South Korea, Article 9-2 of the School Health Act mandates that public educational officials, including elementary school teachers, complete first aid training annually in order to protect students’ health and safety [1]. Accordingly, CPR training for elementary school educators is not optional but a legal and professional responsibility.

Despite this policy framework, current CPR education in schools is often delivered in a uniform, lecture-oriented, and procedure-focused manner, raising concerns about its effectiveness and sustainability [2]. Training is frequently implemented as a one-time, mandatory session aimed at formal completion rather than at meaningful learning, skill retention, or behavioral change. To improve the quality and long-term impact of CPR education, it is essential to move beyond simple knowledge transfer and foster learners’ active engagement and internal motivation [3].

Achievement goal orientation is one of the core constructs associated with learning motivation. It refers to the types of goals that learners pursue when engaging in a task and influences how they allocate effort, persist in the face of difficulty, and interpret success or failure [4,5]. Learners with different goal orientations—such as self- or ego-focused versus task- or mastery-focused—are likely to show distinct patterns of participation and persistence during CPR training. Understanding these patterns is important because CPR education requires not only cognitive understanding but also repeated psychomotor practice.

Immersion (or flow) is another key psychological state relevant to effective CPR training. Immersion refers to a condition in which individuals become deeply absorbed in an activity, experiencing high concentration and intrinsic enjoyment without the need for external rewards [6,7]. Higher levels of immersion have been associated with improved academic achievement, satisfaction, and sustained engagement in learning. Prior studies suggest that immersion is closely linked to achievement goal orientation and self-regulatory behaviors, indicating that promoting immersion can contribute to more positive learning outcomes [8,9,10].

Self-management, in turn, refers to a strategic process in which individuals regulate their own behavior, thoughts, emotions, and environment to achieve desired goals [11,12,13,14]. It is often discussed in relation to self-control or self-regulation and encompasses behaviors such as planning, practice management, interpersonal management, mental control, and physical condition management. Learners with strong self-management skills tend to set goals autonomously, strive to achieve them, and maintain motivation to participate in education, thereby enhancing immersion and performance [15,16,17,18].

Although CPR education for teachers has become more widespread, most previous research has focused on training content, course structure, or quality evaluation of CPR programs. Relatively few studies have examined the psychological and motivational characteristics of the learners themselves—especially elementary school educational officials who are responsible for children’s safety in school settings. In particular, empirical studies that simultaneously consider achievement goal orientation, CPR education immersion, and self-management in this population are lacking.

Therefore, there is a need to investigate how elementary school educational officials’ achievement goal orientations relate to their immersion in CPR education and their self-management competencies. Such evidence can provide a theoretical and practical basis for designing learner-centered CPR programs that enhance engagement, promote continuous practice, and ultimately improve school safety.

### Purpose of the Study

The purpose of this study was to empirically analyze the influence of elementary school educational officials’ achievement goal orientation on their immersion in CPR education and their self-management, with the aim of generating foundational evidence for the development of learner-centered CPR training programs tailored to this population. Specifically, this study examined the relationships among achievement goal orientation, CPR education immersion, and self-management; investigated the effects of achievement goal orientation on CPR education immersion and self-management; and analyzed the extent to which CPR education immersion contributes to self-management among elementary school educational officials.

## 2. Materials and Methods

### 2.1. Participants and Sampling

Participants in this study were elementary school teachers and administrative staff employed in public elementary schools located in Gangwon Province, South Korea. A total of 200 questionnaires were distributed using a convenience sampling method.

According to G*Power 3.1.2 [19], the minimum required sample size for multiple regression analysis (medium effect size = 0.15, α = 0.05, power = 0.95) was calculated to be 119. After excluding 50 questionnaires due to incomplete responses or statistical outliers, 150 valid responses were included in the final analysis.

The survey was administered immediately following the completion of a CPR training session. Participants received a verbal explanation of the study purpose, provided voluntary informed consent, and completed a self-administered questionnaire on site.

#### Description of the CPR Training Program

The CPR training program evaluated in this study was directly based on the American Heart Association (AHA) Basic Life Support (BLS) guidelines and was not intended to assess the effectiveness of a modified or abbreviated curriculum. Although institutional CPR education frameworks in South Korea may allow for extended training durations of up to four hours, the program implemented for the purpose of this study consisted of a standardized two-hour session that integrated both theoretical instruction and hands-on practice.

The theoretical component covered basic life support principles, recognition of cardiac arrest, and activation of the emergency response system. The practical component focused on high-quality chest compressions, airway management, and the use of an automated external defibrillator (AED) using manikins. The training was delivered by certified instructors, including nationally licensed paramedics and emergency nursing professionals with formal CPR instructor qualifications, in accordance with the current international BLS guidelines issued by the American Heart Association (AHA) and the European Resuscitation Council (ERC).

This study did not compare training effectiveness across different program durations; rather, it assessed participants’ achievement goal orientation, immersion, and self-management immediately after completion of a standardized session.

### 2.2. Instruments

A structured questionnaire was used to assess the key variables of the study. It consisted of items on:

- General characteristics (4 items);
- Achievement goal orientation (13 items);
- CPR education immersion (6 items);
- Self-management (15 items).

All items were rated on a 5-point Likert scale (1 = strongly disagree, 5 = strongly agree). The composition of the questionnaire is summarized in Table 1.

### 2.3. Exploratory Factor Analysis and Reliability

To ensure face validity, the questionnaire was reviewed by two CPR education experts and three professors specializing in emergency medical services. Construct validity was examined using exploratory factor analysis (EFA) with principal component extraction and varimax rotation. Items with factor loadings of 0.50 or higher were retained. The reliability of each subscale was assessed using Cronbach’s α, and all scales demonstrated acceptable to excellent internal consistency (Table 2).

The Achievement Goal Orientation scale was adapted from established achievement goal frameworks and validated instruments used in prior research [9,20]. 

The CPR Education Immersion scale was modified from previously validated immersion measures derived from flow theory [11,14]. 

The Self-management scale was adapted from established self-regulation and self-management questionnaires used in prior studies [15,16,17,18].

### 2.4. Data Processing

All statistical analyses were performed using SPSS version 21.0 (IBM Corp., Armonk, NY, USA). A significance level of α = 0.05 was used for all tests.

- Frequency analysis was conducted to examine general characteristics.
- Exploratory factor analysis and Cronbach’s α were used to assess construct validity and internal consistency.
- Pearson’s correlation coefficients were used to examine the relationships among achievement goal orientation, CPR education immersion, and self-management.
- Multiple regression analysis was conducted to identify the effects of achievement goal orientation on CPR education immersion and self-management, as well as the effects of CPR education immersion on self-management.

## 3. Results

### 3.1. Participant Demographics and Professional Background 

Table 3 summarizes the general characteristics of the 150 elementary school educational officials who participated in this study. Participants included both elementary school teachers and administrative staff, as both groups are legally required to complete CPR training and may be responsible for emergency response in school settings. (e.g., initiating CPR/AED use before EMS arrival). This distinction was retained to reflect differences in professional roles and levels of direct student interaction within the school environment.

Females accounted for a larger proportion of the sample (70.7%) compared with males (29.3%). The largest age group was participants aged 20–29 years (44.0%), followed by those aged 50 years or older (25.3%). Regarding job type, 66.0% were elementary school teachers and 34.0% were administrative staff. In terms of professional experience in school settings, the majority of participants had less than five years of experience (60.7%), indicating that many respondents were relatively early in their careers. “Professional experience” refers to total years of employment in school settings for both teachers and administrative staff, not teaching-only tenure.

### 3.2. Correlations Among Achievement Goal Orientation, CPR Education Immersion, and Self-Management

For clarity, Q1–Q8 represent the measured sub-variables of the study constructs: self-goal orientation (Q1), task goal orientation (Q2), cognitive immersion in CPR education (Q3), behavioral immersion in CPR education (Q4), and four domains of self-management—training management (Q5), interpersonal management (Q6), mental management (Q7), and body management (Q8). The full set of questionnaire items and subscale composition are provided in Appendix A for transparency and ease of interpretation. The correlation analysis results are presented in Table 4. All variables showed significant positive correlations with one another. Notably, cognitive immersion demonstrated a very high correlation with behavioral immersion (r = 0.736, *p* < 0.01), indicating a strong association between the two dimensions of CPR education immersion. Additionally, the sub-factors of self-management (training, interpersonal, mental, and body management) showed moderate to high correlations with both types of goal orientation and CPR education immersion. Variance Inflation Factor (VIF) values for all variables were below 10, confirming that multicollinearity was not present.

These correlation patterns suggest that higher levels of mastery- and performance-oriented motivation are associated with greater engagement in CPR education and stronger self-management capacities. In particular, the strong association between cognitive and behavioral immersion indicates that psychological engagement during CPR training is closely linked to active skill practice.

### 3.3. Regression Analysis: Achievement Goal Orientation and CPR Education Immersion 

Multiple regression analysis was conducted to examine the influence of achievement goal orientation on CPR education immersion. As presented in Table 5, the overall model was statistically significant (F = 27.944, *p* < 0.001), explaining 27.5% of the variance in CPR education immersion (R^2^ = 0.275). Both self-goal orientation (β = 0.245, *p* < 0.01) and task goal orientation (β = 0.364, *p* < 0.001) had significant positive effects on CPR education immersion.

### 3.4. Regression Analysis: Achievement Goal Orientation and Self-Management

Table 6 shows the results of the regression analysis examining the influence of achievement goal orientation on self-management. The model was significant (F = 26.355, *p* < 0.001), explaining 26.4% of the variance in self-management (R^2^ = 0.264). Only task goal orientation significantly predicted self-management (β = 0.462, *p* < 0.001). Self-goal orientation did not have a statistically significant effect.

### 3.5. Regression Analysis: CPR Education Immersion and Self-Management

Multiple regression analysis was conducted to examine the effect of CPR education immersion on self-management. As presented in Table 7, the model was significant (F = 26.846, *p* < 0.001), explaining 26.8% of the variance (R^2^ = 0.268). Both cognitive immersion (β = 0.217, *p* < 0.05) and behavioral immersion (β = 0.336, *p* < 0.01) significantly predicted self-management.

## 4. Discussion

This study examined how achievement goal orientation influences CPR education immersion and self-management among elementary school educational officials. By integrating constructs of motivation, immersion, and self-regulated behavior, this study provides foundational evidence for enhancing CPR education effectiveness within school settings. The key findings and their implications are discussed below.

### 4.1. General Characteristics of the Participants

The demographic composition of the participants reflects the typical structure of the elementary school workforce in South Korea, where women comprise the majority of teaching personnel. The large proportion of participants in their 20s and those with less than five years of experience suggests that many educational officials are relatively new to school environments and may have limited exposure to CPR training. This underscores the need for tailored CPR programs that take into account participants’ baseline familiarity, training experience, and learning needs. At the same time, a considerable number of participants had 15 or more years of experience, indicating that CPR training programs must address a wide spectrum of backgrounds. Differentiated instruction—such as novice-friendly modules and advanced skill refreshers—may therefore optimize training effectiveness across experience levels.

### 4.2. Relationships Among Achievement Goal Orientation, CPR Education Immersion, and Self-Management

Both self-goal (performance-oriented) and task goal (mastery-oriented) orientations were positively related to CPR immersion. Notably, self-goal orientation showed its strongest association with behavioral immersion, suggesting that individuals motivated by social comparison may invest greater effort in observable performance during psychomotor training. This finding is consistent with the 2 × 2 achievement goal framework proposed by Elliot and McGregor [9], which posits that performance-oriented goals can temporarily enhance engagement when evaluative contexts are salient.

Previous studies in educational and performance-based training contexts have similarly reported that achievement goal orientation is closely associated with self-management and immersion-related engagement outcomes. [21,22]. Task goal orientation also exhibited robust correlations with self-management domains. This pattern is theoretically coherent with the multiple goal perspective articulated by Pintrich [20], which emphasizes that mastery-oriented learners are more likely to adopt adaptive regulatory strategies across learning tasks. Motivation has also been conceptualized as a key enabling factor that supports learners’ cognitive engagement, persistence, and effective self-regulation in academic contexts [23]. In addition, autonomy-supportive motivational frameworks highlight that intrinsic goal contents promote sustained engagement, persistence, and self-regulation [24]. Collectively, these perspectives help explain why mastery-oriented motivation is closely associated with both deeper immersion and stronger self-management capacities in CPR education contexts.

### 4.3. Effects Identified Through Multiple Regression Analysis

The finding that task goal orientation exerted a stronger effect on CPR education immersion than self-goal orientation aligns with classic motivational theories emphasizing adaptive learning under mastery-oriented conditions. Dweck’s work on motivational processes suggests that mastery goals foster intrinsic interest, resilience, and the use of adaptive learning strategies, which support sustained engagement during skill acquisition [25]. Similarly, mastery-oriented classroom climates have been shown to promote sustained cognitive involvement and strategic learning behaviors, reinforcing the present interpretation [26].

Furthermore, the significant predictive effects of CPR education immersion on self-management can be interpreted through social–cognitive explanations. Self-efficacy theory posits that successful engagement in skill practice enhances learners’ confidence, which in turn facilitates goal setting, effort regulation, and emotional control [27]. From an achievement goal perspective, engagement functions as a key pathway linking motivational orientation to long-term self-regulated outcomes, particularly when learners perceive competence development rather than social comparison as the primary goal [28]. This interpretation is consistent with Zimmerman’s cyclical model of self-regulation, in which performance experiences reinforce self-efficacy and subsequent strategic control processes [29].

The observed pattern that mastery-oriented processes are more supportive of sustained self-management than performance-oriented processes is also consistent with Nicholls’ ability–effort conception [30], which emphasizes effort-based interpretations of competence. Subsequent empirical work has further demonstrated that mastery goals are more strongly associated with persistence and adaptive achievement-related outcomes over time, whereas performance goals tend to be linked to short-term effort under evaluative conditions [31].

Finally, the stronger association between task-goal orientation and cognitive immersion can be understood in light of prior findings showing that mastery-oriented goals are closely related to deep engagement and immersion in learning and performance contexts [32]. This relationship is further supported by Dweck and Leggett’s social–cognitive approach, which underscores the role of mastery goals in fostering intrinsic motivation, adaptive learning strategies, and resilience [33]. At the educational level, schoolwide motivational approaches have similarly emphasized that mastery-oriented climates promote sustained engagement and self-regulation [34]. Prior research has also reported differential effects of mastery versus extrinsic goal orientations on self-regulated learning processes, further supporting the present findings [35]. Taken together, these results suggest that immersion operates as a psychological mechanism through which mastery-oriented motivation is translated into durable self-management in CPR skill acquisition.

In practical terms, a mastery-oriented CPR training framework does not alter the core content of standardized BLS guidelines, but rather modifies how the training is delivered and experienced. This includes emphasizing self-paced hands-on practice, minimizing excessive social comparison, and providing formative feedback focused on individual progress and competence development. Incorporating brief reflective activities, such as structured self-assessment following practice, may further enhance learners’ immersion and self-management during CPR training.

### 4.4. Limitations

Several limitations of this study should be acknowledged. First, the cross-sectional design does not allow causal inferences to be drawn between achievement goal orientation, CPR education immersion, and self-management. Therefore, the observed relationships should be interpreted as associations rather than causal effects. Second, all variables were measured using self-reported questionnaires, which may be subject to social desirability and recall bias. Third, the sample was drawn from a single province in South Korea using convenience sampling, which may limit the generalizability of the findings to other regions or educational contexts. Future studies employing longitudinal designs, objective performance measures, and more diverse samples are warranted to further validate and extend the present results.

## 5. Conclusions

Using post-session survey data from 150 elementary school educational officials, this study found that achievement goal orientation was significantly associated with CPR education immersion and self-management. Specifically, both self-goal (performance-oriented) and task goal (mastery-oriented) orientations were significant predictors of immersion in CPR education, whereas only task goal orientation was a significant predictor of self-management. In addition, both cognitive and behavioral dimensions of CPR education immersion significantly enhanced self-management.

Importantly, these findings do not suggest replacing or revising the existing American Heart Association (AHA) Basic Life Support (BLS) curriculum itself. Rather, they indicate that CPR training programs delivered within standardized BLS guidelines may benefit from pedagogical redesigns that foster mastery-oriented motivation and deeper learner immersion, particularly for elementary school educational officials. In this context, the proposed shift from uniform, compliance-based instruction refers to changes in instructional design and delivery—such as emphasizing hands-on practice, self-paced mastery, and reflective feedback—rather than the development of an entirely separate CPR curriculum.

By incorporating motivational principles into the implementation of standardized CPR training, educators may promote more sustained engagement and more durable self-management behaviors among school personnel. Future research should extend these findings by including participants from diverse regions and occupational contexts and by employing longitudinal or structural modeling approaches that integrate motivational, cognitive, and behavioral components. Qualitative studies using direct observation or interviews may further clarify how mastery-oriented motivation and immersion processes influence post-training self-management in CPR education.

## Figures and Tables

**Table 1:** Composition of Questionnaire.

Variable	Sub-Factor	Number
Achievement Goal Orientation	Self-goal orientation 6	13
	Task goal orientation 7	
Cardiopulmonary Resuscitation Education Immersion	Cognitive commitment 3	6
	Behavioral commitment 3	
Self-management	Training management	15
	Interpersonal management	
	Mental management	
	Body management	
General Characteristics		4
Total		38

**Table 2:** Reliability of Measurement Tools.

Variable	Sub-Factor Reliability		Reliability
Achievement Goal Orientation	Self-goal orientation	0.843	0.885
	Task goal orientation	0.887	
Cardiopulmonary Resuscitation Education Immersion	Cognitive commitment	0.795	0.879
	Behavioral commitment	0.904	
Self-management	Training management	0.810	0.926
	Interpersonal management	0.870	
	Mental management	0.887	
	Body management	0.751	

**Table 3:** General Characteristics of Participants.

General Characteristics		Frequency (n)	Percent (%)
Gender	Male	44	29.3
	Female	106	70.7
Age	20–29	66	44.0
	30–39	23	15.3
	40–49	23	15.3
	≥50	38	25.3
Job Type	Elementary school teacher	99	66.0
	administrative staff	51	34.0
Professional experience	Less than 5 years	91	60.7
	Less than 10 years	10	6.7
	Less than 15 years	11	7.3
	15 years or more	38	25.3

Note: “Professional experience” indicates total years of employment in school settings for both teachers and administrative staff (i.e., not teaching-only tenure).

**Table 4:** Correlations among Achievement Goal Orientation, CPR Education Immersion, and Self-Management.

	Q1	Q2	Q3	Q4	Q5	Q6	Q7	Q8
Self-goal orientation (Q1)	1							
Task goal orientation (Q2)	0.464 **	1						
Cognitive immersion (Q3)	0.373 **	0.494 **	1					
Behavioral Immersion (Q4)	0.473 **	0.409 **	0.736 **	1				
Training management (Q5)	0.366 **	0.433 **	0.382 **	0.486 **	1			
Interpersonal management (Q6)	0.174 *	0.487 **	0.489 **	0.399 **	0.672 **	1		
Mental management (Q7)	0.221 **	0.464 **	0.380 **	0.367 **	0.694 **	0.697 **	1	
Body management (Q8)	0.282 **	0.344 **	0.342 **	0.483 **	0.712 **	0.546 **	0.504 **	1

* *p* < 0.05, ** *p* < 0.01.

**Table 5:** Effects of Achievement Goal Orientation on CPR Education Immersion.

Variable		CPR Education Immersion			
		B	β	t	*p*
Goal achievement orientation	Self-goal orientation	0.234	0.245	3.091 **	0.002
	Task goal orientation	0.418	0.364	4.912 ***	0.000
Constants		3.220		2.661 **	0.009
R^2^		0.275			
F(*p*)		27.944 ***(0.000)			

** *p* < 0.01 *** *p* < 0.001.

**Table 6:** Effects of Achievement Goal Orientation on Self-Management.

Variable		Self-Management			
		B	β	t	*p*
Goal achievement orientation	Self-goal orientation	0.084	0.097	1.210	0.228
	Task goal orientation	0.481	0.462	5.782 ***	0.000
Constants		6.645		6.000 ***	0.000
R^2^		0.264			
F(*p*)		26.355 ***(0.000)			

*** *p* < 0.001.

**Table 7:** Effects of CPR Education Immersion on Self-Management.

Variable		Self-Management			
		B	β	t	*p*
CPR education immersion	Cognitive commitment	0.424	0.217	2.079 *	0.039
	Behavioral commitment	0.503	0.336	3.228 **	0.002
Constants		9.187		10.534 ***	0.000
R^2^		0.268			
F(*p*)		26.846 ***(0.000)			

* *p* < 0.05, ** *p* < 0.01 *** *p* < 0.001.

## Data Availability

The data presented in this study are available upon reasonable request from the corresponding author. The data are not publicly available due to institutional privacy regulations.

## Abbreviations

The following abbreviations are used in this manuscript:

AED	Automated External Defibrillator
AHA	American Heart Association
BLS	Basic Life Support
CPR	Cardiopulmonary Resuscitation
EFA	Exploratory Factor Analysis
ERC	European Resuscitation Council
IRB	Institutional Review Board
KSL	Kids Save Lives
OHCA	Out-of-Hospital Cardiac Arrest
SPSS	Statistical Package for the Social Sciences
VIF	Variance Inflation Factor

## Appendix A. Questionnaire Overview

This table presents representative questionnaire items used to assess each study construct. All items were rated on a 5-point Likert scale (1 = strongly disagree, 5 = strongly agree).

**Table A1:** Representative Questionnaire Items by Construct.

Construct	Sub-Factor	Representative Item
Achievement Goal Orientation	Self-goal orientation	I want to perform CPR skills better than others.
	Task-goal orientation	Improving my CPR skills is more important than comparison with others.
CPR Education Immersion	Cognitive immersion	I was fully concentrated on the CPR training content.
	Behavioral immersion	I actively participated in hands-on CPR practice.
Self-Management	Training management	I manage my practice time effectively during CPR training.
	Interpersonal management	I cooperate well with others during CPR training.
	Mental management	I can control anxiety during CPR practice.
	Body management	I pay attention to my physical condition when performing CPR.
